# Assessment of the Minimal Targeted Biopsy Core Number per MRI Lesion for Improving Prostate Cancer Grading Prediction

**DOI:** 10.3390/jcm9010225

**Published:** 2020-01-15

**Authors:** Guillaume Ploussard, Jean-Baptiste Beauval, Raphaële Renard-Penna, Marine Lesourd, Cécile Manceau, Christophe Almeras, Jean-Romain Gautier, Guillaume Loison, Daniel Portalez, Ambroise Salin, Michel Soulié, Christophe Tollon, Bernard Malavaud, Mathieu Roumiguié

**Affiliations:** 1 Department of Urology, La Croix du Sud Hospital, 52, chemin de Ribaute, 31130 Quint Fonsegrives, France; jbbeauval@gmail.com (J.-B.B.); c_almeras@yahoo.fr (C.A.); gautierjr@hotmail.fr (J.-R.G.); guillaumeloison@gmail.com (G.L.); ambroise.salin@gmail.com (A.S.); tol@club-internet.fr (C.T.); 2 Department of Urology, Institut Universitaire du Cancer Toulouse—Oncopole, 31000 Toulouse, France; marine_lsrde@hotmail.fr (M.L.); bernard.malavaud@me.com (B.M.); roumiguie.m@chu-toulouse.fr (M.R.); 3 Department of Radiology, CHU La Pitié Salpétrière/Tenon, Sorbonne Université, 75005 Paris, France; raphaele.renardpenna@gmail.com; 4 Department of Urology, CHU Toulouse, 31000 Toulouse, France; cecile.manceau2@gmail.com (C.M.); soulie.m@chu-toulouse.fr (M.S.); 5 Department of Radiology, Institut Universitaire du Cancer Toulouse—Oncopole, 31000 Toulouse, France; dportalez@gmail.com

**Keywords:** prostate cancer, radical prostatectomy, upgrading, biopsy, targeted biopsies, MRI, fusion biopsies, systematic biopsies

## Abstract

Background: To study the impact of MRI characteristics and of targeted biopsy (TB) core number on the final grade group (GG) prediction. Materials and Methods: The cohort was 478 consecutive patients who underwent radical prostatectomy (RP) after positive mpMRI (multiparametric magnetic resonance imaging) followed by fusion TB. Endpoints were the upgrading and concordance rates between TB and RP specimens. Results: Upgrading rate after TB was 40.6%. Patients with upgrading had lower PIRADS (Prostate Imaging-Reporting and Data System) scores (*p* < 0.001), smaller lesion size (*p* = 0.017), fewer TB cores (*p* < 0.001), and lower TB density (*p* = 0.015) compared with cases with grade concordance. There was a significant continuous improvement in upgrading rate when TB core number per lesion increased from 56.3% to 25.6% when <2 or ≥5 TB cores were taken, respectively (*p* = 0.002). The minimal TB number per lesion to reduce upgrading risk to approximately 30%was 4 in PIRADS 3, and 3 in PIRADS 4–5 cases. Conclusions: Grade group prediction by TB is significantly improved by higher PIRADS score, larger lesion size, and increased TB per lesion. At least four TB cores should be taken in PIRADS 3 score lesions, whereas three cores seem enough in PIRADS 4–5 cases to improve GG prediction and limit upgrading risk.

## 1. Introduction

Actual prostate cancer guidelines recommend the realization of a multiparametric MRI prior to biopsies [1,2,3,4,5]. The ideal way to target remains unclear (in-bore, cognitive, or software-based fusion; transrectal; or transperineal approach) as well as the ideal targeted biopsy (TB) core number to be taken [6,7]. Moreover, no study has assessed the ideal TB core number for an adequate grade group prediction and to reduce the risk of final upgrading. The minimal TB core number to be taken could be modified according to the MRI characteristics of the lesion in terms of PIRADS (Prostate Imaging-Reporting and Data System) score and number, location, and size of visible lesions, and these stratifications have not been thoroughly evaluated [8]. Finally, the realization of concomitant systematic biopsy (SB) could also influence this ideal TB core number and improve grading prediction. Indeed, SBs are still recommended in addition to TB, aimed at correction of targeting errors and/or false negative areas on imaging, and could limit the interest of a “saturation” TB scheme of the visible lesion [9,10,11,12,13].

How many TB cores should be taken per lesion to reduce the risk of upgrading? How do MRI characteristics (PIRADS score, maximum diameter of lesions, number of lesions) and the concomitant realization of SB impact this TB core number? Given the lack of evidence answering these daily practice questions, we aimed to assess the impact of MRI characteristics (score, size, number of the lesions), and TB core number on final grade group (GG) prediction and upgrading risk in this cohort of consecutive patients undergoing radical prostatectomy (RP) after software-based elastic fusion TB.

## 2. Materials and Methods

### 2.1. Study Population

All subjects gave their informed consent for inclusion before they participated in the study. The study was conducted in accordance with the Declaration of Helsinki, and the protocol was approved by the Ethics Committee of both institutions.

After institutional review board approval, 478 patients underwent a RP for pathologically biopsy-proven prostate cancer after a pre-biopsy positive (PIRADS ≥ 3) multiparametric (mp) MRI. Only initial biopsies were considered (patients undergoing repeat biopsy and control biopsies on active surveillance were excluded). Data were collected into a prospective cohort, and analyses were done in a retrospective manner.

### 2.2. MR Technique

All MRI exams were performed using a 1.5 T clinical system using a 16 channel phased-array torso coil. The MRI protocol included 3DT2w images, diffusion-weighted imaging, and dynamic contrast-enhanced MRI, according to the European Society of Urogenital Radiology guidelines [14,15,16]. The maximal b-value used for diffusion-weighted-imaging was b 2000 s/mm^2^. The mpMRI lesions were scored and reported according to the Prostate Imaging-Reporting and Data System v.2 (PI-RADS) [15]. A suspicious lesion was defined when the PIRADS score ≥ 3.

All patients underwent software-based elastic fusion TB. MRI lesions were submitted to targeted biopsy using real-time transrectal ultrasound (TRUS) guidance via a software registration system with elastic fusion (Koelis^®^ system). At least two targeted cores were sampled by MRI lesion. More targeted cores were permitted at the physician’s discretion, mainly in case of large lesions, or concerns regarding fusion/targeting or access to the lesion (anterior or far apical lesion). RP was performed by high volume surgeons. All imaging and biopsy procedures were performed in two institutions by radiology (two radiologists per center, experience > 5 years in MRI reading) and urology seniors (six urologists, experience in fusion biopsy > 2 years) experienced in prostate cancer diagnosis. Biopsy and RP specimens were evaluated by senior dedicated uropathologists from the two institutions. Grade group was assessed in each separated core. Data from clinical evaluation, biopsy and RP specimens, and follow-up were recorded in a prospective database.

### 2.3. Analyses

The clinical, biological, and pathological findings were assessed in the overall population.

The primary endpoint was the upgrading rate between biopsy and RP grade group (GG), defined by a higher GG in RP specimens compared with that reported on biopsy cores. We analyzed factors associated with this upgrading rate as follows: MRI lesion characteristics (PIRADS score, number of MRI lesions, maximum diameter of the lesion) and TB core number (overall and per lesion). We also assessed an exploratory criterion, TB density, defined by the TB core number divided by the lesion size, in order to evaluate the TB sampling density in MRI lesions (number of TB per mm of lesion size). Other clinicobiological parameters (age, PSAD (PSA density), PSA (Prostate Specific Antigen), prostate volume) were also assessed.

As a secondary endpoint, we evaluated the concordance rate, defined by the same GG between TB and RP specimens (no upgrading, no downgrading).

### 2.4. Statistics

The qualitative data were tested using a chi-square test or Fisher’s exact test as appropriate, and the continuous data were tested using Student’s *t*-test. The Mann–Whitney test was used in cases of no normal distribution. The limit of statistical significance was defined as *p* < 0.05. SPSS 22.0 (Chicago, IL, USA) software was used for analysis.

## 3. Results

Main patient characteristics have already been published in our previous publication [17]. Median age and PSA were 65.6 years and 8 ng/mL. Pre-biopsy imaging classified MRI lesions as PIRADS score 3, 4, and 5 lesions in 20.5%, 49.8%, and 29.7% of the cohort, respectively. One MRI lesion was reported in 71.7% of the cases with a median number of 1.36 (median 1). Median MRI lesion size was 11 mm. The median number of positive cores was five (two on TB, three on SB). Grade group predicted by TB was upgraded in 40.6% of cases in RP specimens. This rate decreased to 31.8% by adding systematic biopsies to TB. TB adequately graded cancer in 45.2% of cases. GG2 cancers on TB were upgraded to GG3 and GG4–5 cancer in RP specimens in 31.6% and 3.2% of cases, respectively. Grade group correlations between TB and RP are reported in Table 1.

Correlations between upgrading rate and quantitative variables are reported in Table 2. No significant association was seen concerning age, PSA, PSAD, or prostate volume. MRI characteristics influenced the risk of upgrading. Indeed, PIRADS score, maximum diameter of the lesion, TB core number, and TB density were significantly associated with upgrading after TB. Upgrading cases had lower PIRADS scores (*p* < 0.001), smaller lesion size (*p* = 0.017), fewer TB cores in total and per lesion (*p* < 0.001), and lower TB density (*p* = 0.015) compared with cases with GG concordance between TB and RP specimens.

**Figure 1:**
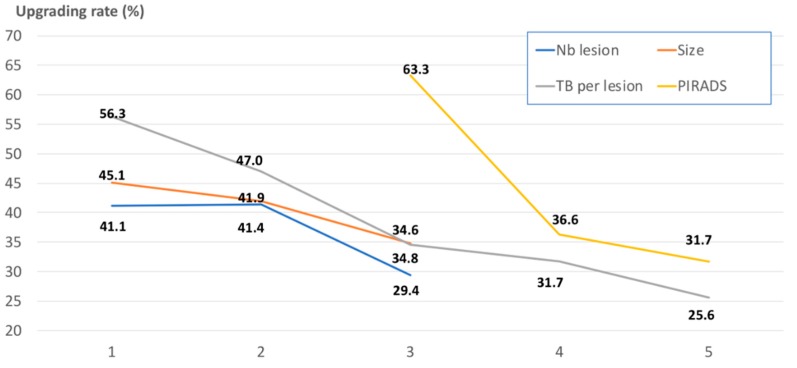
Illustrations of the upgrading rates after targeted biopsies (TB) (in %; on the vertical axis) according to different pre-biopsy parameters: PIRADS (Prostate Imaging-Reporting and Data System) score (yellow line; 3 vs. 4 vs. 5 on the horizontal axis), MRI (magnetic resonance imaging) lesion size (orange line; 1: <10 mm; 2: 10–15 mm; 3: ≥15 mm on the horizontal axis), TB core number (grey line; 1 vs. 2 vs. 3 vs. 4 vs. ≥5 on the horizontal axis), and number of MRI lesions (blue line; 1 vs. 2 vs. ≥3 on the horizontal axis).

MRI characteristics and TB core number were then assessed as qualitative variables, as shown in Table 3 and illustrated in Figure 1. The risk of upgrading from TB to RP specimens significantly decreased from 63.3% in PIRADS 3 to 31.7%–36.6% in PIRADS 4–5 cases (*p* < 0.001). This difference remained when SBs were associated with TB. No significant difference of upgrading rate was reported between one or two MRI lesions (41%). Upgrading rate progressively and non-significantly decreased with increased MRI size, from 45.1% in <10 mm lesions to 34.8% in ≥15 mm lesions. There was a significant continuous improvement in upgrading rate when TB core number per lesion increased. Upgrading rate was 56.3% when fewer two TBs per lesion were used, and decreased to 47.0%, 34.6%, 31.7%, and 25.6% when two, three, four, and over five TB cores per lesion were sampled (*p* = 0.002). There was a non-significant trend suggesting that only a high TB density (≥0.40 TB/mm) did have a positive impact on decreased upgrading rate.

The same trends were reported when assessing the concordance between TB and RP specimens. The concordance rate significantly increased from 33.7% to 48.2% between PIRADS 3 and 4–5 lesions (*p* = 0.015), and from 37.7% to 52.2% between <10 and ≥15 mm lesions (*p* = 0.025). The concordance rates were 39.4%, 46.8%, and 53.0% when one to two, three to four, and over five TBs per lesion were taken, respectively (*p* = 0.024).

We then explored the impact of TB core number per lesion on upgrading rate according to the PIRADS score (Table 4). Taking one, two, or three cores did not modify the upgrading rate in PIRADS 3 cases (66%). The rate significantly decreased to one-third only when at least four TB cores were taken. In PIRADS 4–5 cases, taking three cores per lesion appeared enough to improve GG prediction (from 41.0% to 29.0%), without improvement when more TB cores were used.

When assessing the impact of MRI characteristics in multivariable analysis (Table 5), PIRADS score was the only independent factor predictive for upgrading after TB (*p* < 0.001). The risk of upgrading was decreased by 2.9- and 3.7-fold in PIRADS 4 and 5 lesions, respectively, compared with PIRADS 3. When using a combination of TB and SB, PIRADS score (*p* < 0.001) and TB core number per lesion (*p* = 0.023) were the two independent predictive factors for upgrading.

## 4. Discussion

Pre-biopsy MRI followed by TB if positive is recommended by actual prostate cancer guidelines [1]. The imaging-based strategy has proven to be meaningfully relevant in terms of clinically significant prostate cancer detection by high-level studies [2,3,4,5]. MRI targeting might also improve the prognostic assessment and the prediction of grade group of the disease. Nevertheless, some concerns remain, including the ideal TB core number to be taken per lesion. In the vast majority of published trials, two to three cores were sampled per visible lesion [2,3,4,5]. In other studies, the TB core number was decided at physician’s discretion, and no clear consensus exists. Lu et al. recently found that sampling of five cores missed substantially fewer cancers than two cores [7]. However, they did not assess that ideal core number as a function of MRI lesion characteristics such as maximum diameter of the lesion, number, and PIRADS score. Moreover, the impact of TB core number on final GG prediction (grade in RP specimens) has not been evaluated, although the accuracy of grading assessment strongly impacts treatment decision-making.

In the present study, all patients underwent a RP after a pre-biopsy positive MRI followed by a combined biopsy scheme (TB and SB). The same fusion system was used in all patients to ensure the reproducibility of the technique within the cohort. This led to an acceptable rate of upgrading between TB and RP specimens of 40%, mainly in GG1 and GG2 disease. Indeed, very few high-grade (GG4–5) disease cases were missed by TB (3%).

Does the TB core number impact on this upgrading rate? We found an incremental utility of performing additional TB cores for improving grading prediction and reducing the risk of upgrading. This risk ranged from 56.3% when fewer than two TBs per lesion were taken to 47.0%, 34.6%, 31.7%, and 25.6% when two, three, four, and over five TB cores per lesion were sampled.

The risk of upgrading was also significantly influenced by two main imaging characteristics: PIRADS score and maximum diameter. No difference was reported between one and two lesions (representing 95% of the overall cohort) suggesting that the TB core number should not be adapted as a function of the number of lesions seen on MRI. Upgrading rate was lower (29.3%) when three or four lesions were seen on MRI, but the number of cases was insufficient to draw any strong conclusion (*n* = 24). The concordance rate between TB and RP was meaningfully improved in PIRADS 4–5 lesions (almost 50%) compared with that found in PIRADS 3 lesions (one-third). This could suggest a need for more extended TB sampling in PIRADS 3 lesions. Indeed, we found that the minimal TB number per lesion was four in PIRADS 3 and three in PIRADS 4–5 cases to reduce upgrading risk to approximately 30%. Thus, at least one supplementary TB core was required in PIRADS 3 lesions to obtain the same upgrading rate that was observed in PIRADS 4–5 lesions.

MRI characteristics should guide our decision as to the number of TBs to sample. Indeed, clinical (age, prostate volume) and biological (PSA, PSAD) features did not significantly influence the risk of upgrading. A more extended TB sampling should be preferred in small lesions with a PIRADS 3 score. We also explored the concept of TB density, defined by the TB core number in function of the lesion size. Independently of the PIRADS score, upgrading rate was significantly decreased when TB density was superior to 0.40, meaning at least four cores per 10 mm. An ideal TB scheme should include two cores for sampling a 5 mm, four cores for a 10 mm, and six cores for a 15 mm lesion. Confirmatory analyses are needed to confirm these findings and the adaptation of TB core number according to MRI lesion size.

The usefulness of concomitant SB in case of MRI-targeted biopsy strategy remains debatable. However, the failure rate of the imaging-based strategy is not negligible, in terms of lesion detection rate by imaging and of targeting errors. Recent studies have evaluated that the failure rate of the imaging-guided pathway for detecting index lesions can be estimated at 20% [12]. Indeed, potential sources of error can arise from each step of the imaging-based strategy and may explain the remaining inaccuracy of TB for both detection and accurate prognostic evaluation [11]. Several studies have suggested that TB and SB techniques are complementary and should be offered together, given their variable performances depending the tumor location and other factors [16,18]. Thus, to date, SBs are still recommended in addition to TBs in the first set of biopsies [1]. The realization of concomitant SB might impact the GG prediction and decrease the upgrading rate. In the present study, we found that concomitant SB improved GG prediction and decreased upgrading rate by approximately 10%, whatever the MRI characteristics of the visible lesions. Interestingly, upgrading rates between TB alone and TB + SB schemes were comparable (one-quarter) when at least five TBs were used. A plateau seemed to be reached when at least four cores per lesion were taken in addition to SB, suggesting that five TB cores per lesion might not be necessary if systematic sampling were concomitantly done. Our findings suggested that even if we obtained three or four cores in TB according to different PIRADS score, the upgrading rates would remain high (approximately 30%) which further stresses the need to add SB in order to reduce upgrading at final pathology.

Among the limitations of our study, we would like to emphasize that all analyses were performed retrospectively. Moreover, we did not prospectively report the exact location of the MRI lesions. Thus, we were not able to assess how the location impacted on concordance/upgrading rate. Several studies have suggested that the sampling of anterior lesions could be less accurate than that reported in peripheral lesions, and could lead to a higher risk of upgrading [16]. However, Lu et al. recently noticed that the detection of significant cancer by TB did not differ by tumor location [7]. We used a standard transrectal route for all prostate biopsies; however, a transperineal TB approach might be relevant for these anterior tumors to improve both detection and GG prediction [19]. Experience strongly matters when applying the imaging-based biopsy strategy in terms of both MRI reading and lesion targeting, and this could introduce some biases when assessing the precision of TB. Nevertheless, all radiologists and biopsy operators involved in our study were highly experienced in computer-based fusion devices, and beyond their learning curves prior the beginning of the study period. The same fusion computer-assisted software was used in the two institutions, which reduced interpretation biases. Moreover, this elastic registration system has been proven to improve precision for targeting and to be correlated with improved detection of clinically significant PCa compared with cognitive fusion method [19,20]. We also analyzed the grading concordance rate between biopsy and RP specimens according to the center. The concordance rate for TB (42.2% vs. 47.8%) as well as for SB (32.3% vs. 38.4%) did not differ significantly according to the institution. Data were collected from one academic and one non-academic center. However, the risk of upgrading between TB and RP specimens was comparable between centers (*p* = 0.860).

## 5. Conclusions

Grade group prediction by TB was significantly improved by higher PIRADS score, larger lesion size, and increased TBs per lesion. We found an incremental utility of performing additional TB cores for improving grading prediction and reducing the risk of upgrading. At least four TB cores should be taken in PIRADS 3 score lesions, whereas three cores seemed enough in PIRADS 4–5 cases to improve GG prediction and to limit upgrading risk to 30%. Concomitant SB improved GG prediction and decreased upgrading rate by approximately 10%, whatever MRI characteristics of the visible lesions (PIRADS score, number and size of lesions).

## Figures and Tables

**Table 1:** Correlations between grade groups between TB and RP specimens.

Grade Group on TB	Grade Group on Final Pathology (RP Specimens)				
	1	2	3	4–5	Total
No cancer	7	35	9	1	52
1	2	39	9	3	53
2	4	136	68	7	215
3	1	26	62	8	97
4–5	0	6	29	26	61
Total	14	242	177	55	478

**Table 2:** Correlations between clinicobiological features, MRI characteristics (size, score, number of the lesions), TB core number, TB density, and the presence of upgrading after targeted biopsies (TB).

	Upgrading Rate after TB		
	No *n* = 284	Yes *n* = 194	*p*-Value
Age (mean), years	65.1	64.6	0.410
PSA (mean), ng/mL	10.3	10.6	0.719
PSAD (mean), ng/mL/gram	0.27	0.23	0.297
Prostate volume (mean), mL	48.1	52.0	0.121
PIRADS score (mean)	4.2	3.9	<0.001
No MRI lesions (mean)	1.37	1.34	0.632
MRI lesion size (mean), mm	12.7	11.	0.017
No TB cores(mean)	4.1	3.2	<0.001
No TB per lesion (mean)	3.1	2.6	<0.001
TB density (mean)	0.36	0.30	0.015

**Table 3:** Upgrading rates after targeted biopsies (TB) according to MRI characteristics (size, score, number of the lesions), TB core number, and TB density.

	Number	Upgrading Rate (%) after TB
PIRADS score:		
PIRADS 3	98	63.3
PIRADS 4	238	36.6
PIRADS 5	142	31.7
*p* value		<0.001
MRI lesions:		
1	343	41.1
2	111	41.4
3–4	24	29.3
*p* value		0.504
MRI lesions size (mm):		
<10	131	45.1
10–15	223	41.9
>15	124	34.8
*p* value		0.254
TB core number:		
2	188	50.0
3	65	43.1
4	125	36.0
5	100	27.0
*p* value		0.001
TBs per lesion:		
1	48	56.3
2	202	47.0
3	81	34.6
4	104	31.7
5 or more	43	25.6
*p* value		0.002
TB density:		
<0.20	117	44.4
0.20-0.40	238	42.7
≥0.40	123	34.2
*p* value		0.229

**Table 4:** Impact of the targeted biopsy (TB) core number on upgrading rates stratified by the PIRADS score.

	Upgrading Rate (%) after TB
*PIRADS 3* (*n* = 98) TB per lesion:	
1–2	66.2
3	66.7
4 or more	33.3
*PIRADS 4–*5 (*n* = 380) TB per lesion:	
1–2	41.0
3	29.0
4 or more	29.7
*p*-value	0.002

**Table 5:** Regression multivariable analysis assessing the MRI characteristics correlated with the upgrading rate after TB.

	Upgrading Rate (%) after TB
PIRADS score:	
PIRADS 3	Ref. (Reference)
PIRADS 4	0.34 (0.20–0.57)
PIRADS 5	0.27 (0.13–0.54)
*p*-value	<0.001
MRI lesions:	
1	Ref.
2	1.29 (0.77–2.15)
3–4	1.12 (0.41–3.01)
*p*-value	0.620
MRI lesion size (mm):	
<10	Ref.
10–15	0.77 (0.45–1.32)
>15	0.91 (0.40–2.04)
*p*-value	0.596
TBs per lesion:	
2	Ref.
3–4	0.79 (0.42–1.46)
5 or more	0.57 (0.27–1.18)
*p*-value	0.316
TB density:	
<0.20	Ref.
0.20–0.40	0.90 (0.44–1.47)
≥0.40	0.81 (0.33–2.44)
*p*-value	0.725
